# X-ray Dose Rate and Spectral Measurements during Ultrafast Laser Machining Using a Calibrated (High-Sensitivity) Novel X-ray Detector

**DOI:** 10.3390/ma14164397

**Published:** 2021-08-05

**Authors:** Philip Mosel, Pranitha Sankar, Jan Friedrich Düsing, Günter Dittmar, Thomas Püster, Peter Jäschke, Jan-Willem Vahlbruch, Uwe Morgner, Milutin Kovacev

**Affiliations:** 1 Institute of Quantum Optics, Leibniz Universität Hanover, 30167 Hannover, Germany; sankar@iqo.uni-hannover.de (P.S.); morgner@iqo.uni-hannover.de (U.M.); kovacev@iqo.uni-hannover.de (M.K.); 2 Laser Zentrum Hannover e. V., 30419 Hannover, Germany; j.duesing@lzh.de (J.F.D.); t.puester@lzh.de (T.P.); p.jaeschke@lzh.de (P.J.); 3 Steinbeis Transfer Center Technology Consultancy and Development, 73433 Aalen, Germany; dittmar-transfer@t-online.de; 4 Institute of Radioecology and Radiation Protection, 30419 Hannover, Germany; vahlbruch@irs.uni-hannover.de

**Keywords:** X-ray emission, micromachining, dose rate, X-ray spectrum, ultrafast laser

## Abstract

Ultrashort pulse laser machining is subject to increase the processing speeds by scaling average power and pulse repetition rate, accompanied with higher dose rates of X-ray emission generated during laser–matter interaction. In particular, the X-ray energy range below 10 keV is rarely studied in a quantitative approach. We present measurements with a novel calibrated X-ray detector in the detection range of 2–20 keV and show the dependence of X-ray radiation dose rates and the spectral emissions for different laser parameters from frequently used metals, alloys, and ceramics for ultrafast laser machining. Our investigations include the dose rate dependence on various laser parameters available in ultrafast laser laboratories as well as on industrial laser systems. The measured X-ray dose rates for high repetition rate lasers with different materials definitely exceed the legal limitations in the absence of radiation shielding.

## 1. Introduction

Since the late 1960s, laser has evolved from a laboratory curiosity to a sophisticated industrial tool. Today, the peak-intensity of high-power laser systems can be used in a controlled way to serve as a machining tool for many metallurgical applications, e.g., welding, cutting, drilling, and surface hardening [1]. The main advantage of laser machining is that it generates a higher quality product with minimal deformations or damage at a faster rate, which is a critical manufacturing need [2].

Recently, it has been demonstrated that ultrashort laser machining can be accompanied with an X-ray radiation at high repetition rates, if the peak intensity exceeds 10^13^ W/cm^2^ [3,4,5,6]. Industrial laser sources used for materials processing typically have pulse durations in the range of pico- to femtoseconds. With these pulse durations, it is straight-forward to generate the above-mentioned intensity. With such high laser intensity, the electron temperatures in the produced plasma approach several keV, resulting in bremsstrahlung emissions in the keV X-ray regime [7]. The laser energy transferred to a plasma electron, depends on the mechanism dominating the laser plasma interaction. If the kinetic energy of the plasma electrons becomes high enough, characteristic X-ray radiation can also be generated.

In recent publications, it has been shown that during ultrafast laser machining at high repetition rates of several 100 kHz, the amount of emitted X-ray radiation can exceed the regulatory exposure limits for members of the public [8]. Low X-ray doses per pulse can accumulate through high repetition rates and exceed radiation protection limits. Therefore, a detailed study of the influence of laser and the material parameters used for measuring the X-ray dose rate is crucial to assess appropriate safety measures. Legall et al. [9] measured the spectral X-ray emission and dose rates (Ḣ′(0.07)) for different target materials using an ultrashort-pulse laser up to maximum peak intensity 2.6 × 10^14^ W/cm^2^. They showed that the unwanted emission of X-ray radiation during ultrashort pulse laser processing of materials in air is commonly observed. These results were confirmed by Behrens et al. (2019) under similar experimental conditions [10]. Also, an analytical model was recently presented by Weber et al. (2019) to estimate the expected X-ray dose when laser machining with ultrashort-pulse lasers under industrial conditions up to an intensity 7 × 10^14^ W/cm^2^ [4].

On the other hand, no work has been published for the X-ray emission during industrial machining, in the moderate intensity regime of 10^14^–10^16^ W/cm^2^ with high repetition rate lasers. One of the reasons is that until now there are no commonly available spectrometer with an immediate readout for measuring dose rates with high sensitivity. X-ray emission ranging from a few eV to about 10 keV occurs when processing most of the materials such as stainless steel, copper, various alloys, and ceramics [10]. This X-ray radiation can cause skin cancer hence, this emission range requires more attention. In this paper, we investigate the X-ray emission from different alloys, metals, and ceramics with two high repetition rate lasers. We use a novel calibrated X-ray detector for the measurements which provides a high sensitivity over 2–20 keV range. Experimental methods employed for the determination of the dose rates and the spectral analysis of the different alloys, metals and ceramics is presented. We also compare the dosimeter OD-02 with the novel spectrodosimeter Silix lambda (engineering office Prof. Dr.-Ing. Günter Dittmar) under identical experimental conditions.

## 2. Materials and Methods

### 2.1. Optical Setup

The experiments are performed using two different laser systems with parameters noted in Table 1. The laser beam is focused onto the sample by means of a plano-convex lens/f-theta scanning objective with a focal length of 60 mm. The angle of incidence of the laser beam is perpendicular to the sample plane. The laser intensity varies from 10^13^–10^16^ W/cm^2^. The average power of the pulse is simultaneously measured at one arm from a beam splitter.

**Figure 1:**
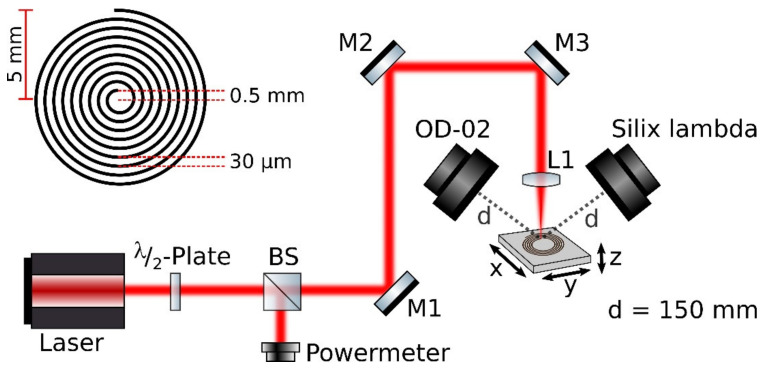
The schematic experimental setup is shown here. M1, M2, M3—Dielectric mirrors; L1—Plano-convex lens (f = 60 mm); BS—beam splitter. The schematic image of the spiral patterning on the material formed during the laser micromachining is shown in the left top. A power meter is used to verify the laser power used for the laser processing.

For our measurements we use different metals like titanium, molybdenum, iron, copper, nickel, zinc, and tungsten. In addition, we process ceramics like ZrO_2_, Si_3_N_4_, and Al_2_O_3_. Moreover, brass, stainless steel, NiCuZn, and NiCrFe alloys have been exposed to intense laser radiation. The percentage composition of alloys can be found in Table 2. The sample is positioned using a motorized three axis stage which is used for ultrafast machining in laboratories for the 1 kHz system and a scanner for the 755 kHz system (Figure 1). To exclude polarization dependence, a spiral with a line spacing of 30 µm is written. The minimum inner diameter is 1 mm. The laser turned-on time is 2.45 s per spiral and turned off for 4 ms during going back to start position. The outer diameter was chosen depending on the repetition rate so that the total duration of the writing process is about 49 s. The distance between pulse to pulse is 1.3 µm for both lasers.

Two different detectors are used for the local dose rate (Ḣ′(0.07)) measurements. A calibrated compact portable ion chamber dosimeter (STEP OD-02, PTW Freiburg, Freiburg im Breisgau, Germany) is used for skin dose measurements Ḣ′(0.07) at a distance of 150 mm from the processing point at an angle of 45° with respect to the laser line. At the same time, we use a calibrated spectrodosimeter Silix lambda, with the same measurement distance and angle, to obtain the X-ray spectral and dose rate information. The detailed description of this calibrated X-ray detector is given in the next section.

**Figure 2:**
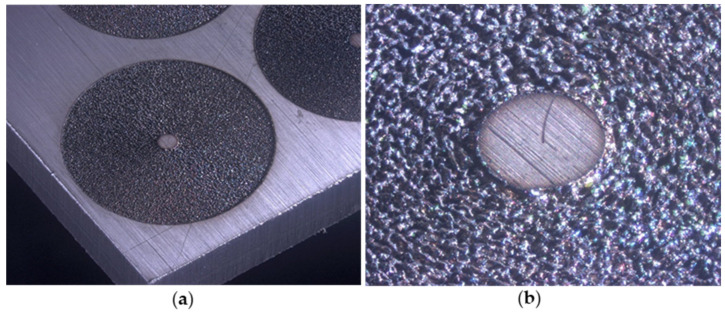
Shown is (**a**) the spiral after laser processing by the Coherent Monaco laser at 4.2 × 10^14^ W/cm^2^, (**b**) a detailed view from the structure.

Figure 2 depicts the typical appearance of the material surface after processing of 20 spirals with the 755 kHz laser system with an irradiation of 4.2 ± 10^14^ W/cm^2^. It should be noted that during this experiment the laser parameters are not optimized for surface quality. Due to high pulse overlap and high average power heat the surface shows formation of resolidified material, indicating that heat accumulation occurred during the laser process. No predominant directional structures are observed in any sample.

### 2.2. Silix Lambda X-ray Spectrodosimeter

The Silix spectrodosimeter is especially developed by the engineering office Prof. Dr.-Ing. Günter Dittmar for measuring X-ray radiation during material processing with ultrashort pulse lasers and to evaluate the X-ray emission with a spectral resolution of 0.08 keV. The Silix spectrodosimeter is calibrated on a reference radiation source of the Physikalisch-Technische Bundesanstalt (PTB) in Braunschweig, Germany using comparative measurements with standardized X-ray spectra.

The working principle of this spectroradiometer is based on the photoelectric effect to detect X-rays. The sensor is a silicon image chip with up to 8 million pixels. The pulsed X-ray radiation generates electrical charge carriers in the silicon chip, which are evaluated by a fast microcontroller. When an image pixel is hit by an X-ray photon, a small current is generated. The greater the energy of the X-ray photon, the greater the photocurrent. Special signal processing separates the photocurrent from interference currents. A new feature is the patented self-monitoring of the device in order to guarantee the proper functioning of the Silix X-ray detector. The equivalent dose rates relevant for radiation protection are calculated internally in the device:

- Directional dose equivalent rate for the skin Ḣ′(0.07).
- Directional dose equivalent rate for the eyes Ḣ′(3).
- Ambient dose equivalent rate Ḣ*(10).

The energy spectrum of the photon irradiance is recorded in 235 channels ranging from 2–20 keV. It is stored and displayed as a curve with a unit of photons (keV cm^2^ s). For measurement at very low irradiances (small dose rate), any number of individual spectra can be averaged. In this way, at photon energies between 2–10 keV—the most important range for laser-induced ionising radiation—dose rates as low as about 0.1 µSv/h can be displayed. In the range of up to 45°, the angular dependence of the measurement signal deviates less than 5% from the cosine function. The measuring sensor has an area of 0.1 cm^2^ and thus enables homogeneous irradiation even at a very short distance of about 2 cm to the point of impact of the laser beam. With these features, the Silix lambda X-ray monitor can also be used in safety-critical monitoring tasks for laser processing applications.

## 3. Results and Discussion

The mechanism generating X-rays during ultrafast laser interaction with materials has been described elsewhere [3,4,8]. Under experimental conditions, high-kinetic-energy electrons would directly collide against electrons in atoms inner shell orbitals, resulting in the emission of characteristic lines for e.g., k-α, k-β line emissions along with a bremsstrahlung mechanism. The X-ray photon flux generated from the laser produced plasma strongly depends on the number of laser photons absorbed. This X-ray photon flux depends on two factors: the initial interaction of material with the laser and subsequent plasma electron heating [11]. Thus, X-ray generation is strongly dependent on laser and material parameters.

Here, we investigate the X-ray emission during ultrashort pulse laser machining of materials which are commonly used in scientific laboratories and laser-based fabrication industries. The intensity is varied in the experiment by rotating a half wave plate in the optical beam path. The results of these experiments are presented in the following sections.

### 3.1. Dose Rates

**Figure 3:**
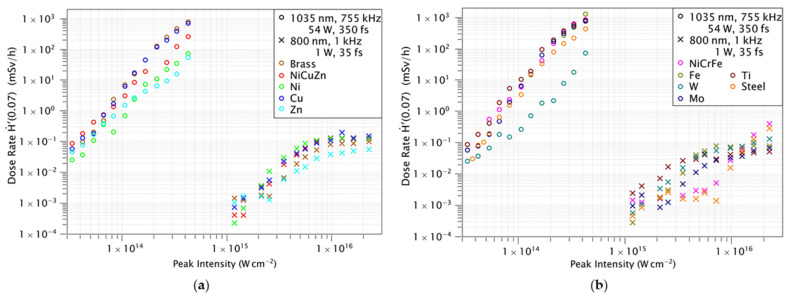
Measured dose rates Ḣ′(0.07) in dependence on the different target material and the incident laser peak intensity listed as: (**a**) Brass, and NiCuZn alloy and its composition metals Ni, Cu, and Zn are investigated using the ionization chamber dosimeter OD-02 at a distance of 150 mm from the interaction zone in air. The laser intensity was varied between 5 × 10^13^ and 5 × 10^16^ W/cm^2^ by tuning the laser pulse energy for two different lasers. (**b**) NiCrFe and stainless steel alloys and its composition metals as well as W, Mo, and Ti are plotted in the same experimental conditions.

The maximum X-ray local dose rates measured for the Monaco (Coherent Inc., Santa Clara, CA, USA) and Dragon (KMLabs, Boulder, CO, USA) lasers in mSv/h as a function of the laser peak intensity using the OD-02 is shown in Figure 3. For the dose rate measurements, three ceramics (Al_2_O_3_, Si_3_N_4_, ZrO_2_), four alloys (brass, steel, nickel brass, NiCrFe) and three metals (titanium, molybdenum, and tungsten) are used with the two laser systems.

In the case of the Coherent Monaco laser system, the dose rate increases with the laser intensity for all materials. Thus, the dose rate reaches 1 Sv/h at the maximum laser intensity.

This corresponds to a maximum X-ray dose per laser pulse of about 0.37 nSv. At an intensity of 4.2 × 10^14^ W/cm^2^, Iron and Molybdenum show the highest dose rate for metals. Among alloys, the highest dose rate is observed in NiCrFe and among ceramics in Si_3_N_4_. We notice a power dependence for the dose rate with the laser peak intensity Ḣ′(0.07) ∝ I^5^.

The maximum dose rate for the KMLabs Dragon 1 kHz system is of 0.3 mSv/h which is significantly lower in comparison with the Coherent Monaco laser. This is about 22% of the dose per laser pulse (0.08 nSv) compared to the 755 kHz system. Despite the higher pulse energy of the KMLabs Dragon laser, we obtained a 4.6 × higher dose rate from the Coherent Monaco. Several parameters—such as central wavelength, pulse duration, spot size, and multi-pulse effects—contribute to this difference in X-ray dose rate. In particular, the bigger focal diameter of the Coherent Monaco laser leads to a 3.4 × larger illuminated area on the surface, while the longer wavelength allows for higher accelerations of the electrons in the plasma and thus contributes to the increase in dose rate. These two parameters could be primarily responsible for such difference in the dose rate measured [12]. Laser absorption to the material has a direct dependence over the wavelength of the laser. If the wavelength is low, the conversion efficiencies of laser photons to the plasma is reduced and it effectively reduces the X-ray dose rates [13]. The material properties have an influence on the conversion efficiency and thus on the resulting dose rate. Additionally, the high repetition rate of the Coherent Monaco laser system may contribute to the higher dose per pulse compared to the 1 kHz system. At high repetition rates heat accumulation in the material can reduce ablation thresholds [14] and increase material removal rates [15]. On the other hand, König et al. [16] have observed that hot material ejected by laser ablation of metals can be present up to several microseconds after the laser pulse. In [16] it is concluded that the material plume possibly consists of metal vapor and droplets which may lead to shielding effects at a repetition rate of some hundreds of kHz and beyond. Thus, it is likely that in our experiments with the 755 kHz laser system particle shielding is present and plasma shielding cannot be completely ruled out. Coupling a considerable amount of laser energy into a particle cloud or plasma of previous ablation events may contribute to an even stronger X-ray emission compared to low repetition rate laser ablation. When processing materials with the KMLabs Dragon laser, Copper has the highest dose rate for metals compared to the Coherent Monaco laser, while NiCrFe has the highest dose rate for alloys.

To understand the physical process of X-ray generation in laser materials processing, the fundamental mechanisms that result in X-ray generation during laser-plasma interaction must be studied. Two different mechanisms are needed to be considered for the generation of X-rays in the intensity range of 10^12^–10^16^ W/cm^2^, namely collisional absorption and collisional less absorption [17,18,19]. The plasma is heated via ‘collisional’ absorption (inverse bremsstrahlung), in which electrons disperse the kinetic energy absorbed from the laser radiation field through collisions with other electrons and ions in the plasma. Through this mechanism, hot electrons are generated and these electrons decelerate in the vicinity of ions and produce bremsstrahlung X-rays. These hot electrons produced in the plasma scale with intensity via inverse bremsstrahlung, and this ultimately results in higher X-ray emission and dose rates which is given by, T_h_ ∝ (Iλ^2^)^α^ with T_h_ hot electron temperature, I, λ intensity and wavelength of the laser, respectively [20].

It is commonly accepted, that at intensities below 10^13^ W/cm^2^ collisional absorption dominates the laser plasma interaction [17,18,19]. In this regime, the X-ray dose rate is power dependent on the intensity. However, for an intensity regime above 10^15^ W/cm^2^, a mechanism where the laser electric field resonantly excites plasma waves, close to the surface of the over-dense plasma region, takes place. Here the laser field cannot propagate which is commonly referred to as ‘collision-less’ absorption regime and hence the dependency of the dose rate is weaker and almost saturates at a plateau. At this point the intensity depends on the dose rate as (Iλ^2^)^1/3^ which confirms that resonance absorption dominates in this intensity regime.

When we compare the parameters of the used laser systems such as the wavelength, pulse duration, pulse energy and repetition rate, we observe a strong influence on the X-ray dose rate. At high laser pulse repetition rates in the hundreds of kHz range, the accumulated number of pulses over a time is very high. Hence, the heat accumulation on the target is increasing. This will support the plasma generation to produce more hot electrons and can explain the increase of X-ray doses at higher repetition rate.

In addition, the conversion efficiency of X-ray generation should be considered to estimate the dose rates, which is defined as the ratio of the energy emitted as X-rays to the energy of the incident laser pulse. This conversion efficiency depends critically on the target geometry in which the laser–plasma interaction takes place, material absorption coefficient, thermal conductivity and laser parameters—e.g., the laser pulse duration, wavelength, and intensity [11].

When we compare the X-ray emission from alloys with the emission from its composition metals, the dose rates of alloys can be increased or suppressed according to the dose rates of its composite metals. Take brass as an example, its dose rate scales similar to its composition metal of copper. On the contrary, the NiCuZn dose rate is suppressed due to its nickel composition. Similarly, in the case of the NiCrFe alloy, the dose rate follows that of iron and chromium rather than scaling with the Nickel dose rates. Therefore, the alloy dose rate is not completely relying on the dose rates of its metal compositions. However, these first observations are still preliminary and further work is needed in order to fully understand the dependencies on the materials.

The measured X-ray dose rates shown in Figure 2 exceed the skin dose of 20 mSv per year already at an exposure time of 80 s [21]. The maximum dose rate Ḣ′(0.07) measured at the highest intensity is 899 mSv/h and 0.40 mSv/h for Coherent Monaco and KMLabs Dragon lasers, respectively. So, it is recommended to take necessary measures to ensure personal safety during the micromachining process. Also, we should find the working distance from the interaction zone with an acceptable dose rates and proper shielding for a safe working environment. The permissible occupational radiation exposure should be monitored before each experiment.

### 3.2. Spectra

**Figure 4:**
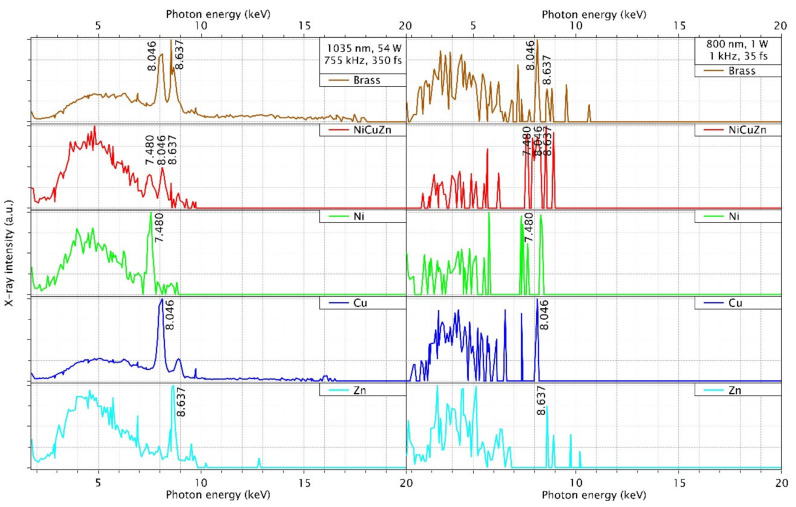
X-ray spectra measured for the different target material and the incident laser peak intensity listed as brass and NiCuZn alloy and its composition metals Ni, Cu, and Zn are investigated using a Silix lambda detector at a distance of 150 mm from interaction zone in air at an intensity 4.2 × 10^14^ W/cm^2^ (**left**) and 2.3 × 10^16^ W/cm^2^ (**right**) respectively for the above mentioned two lasers.

**Figure 5:**
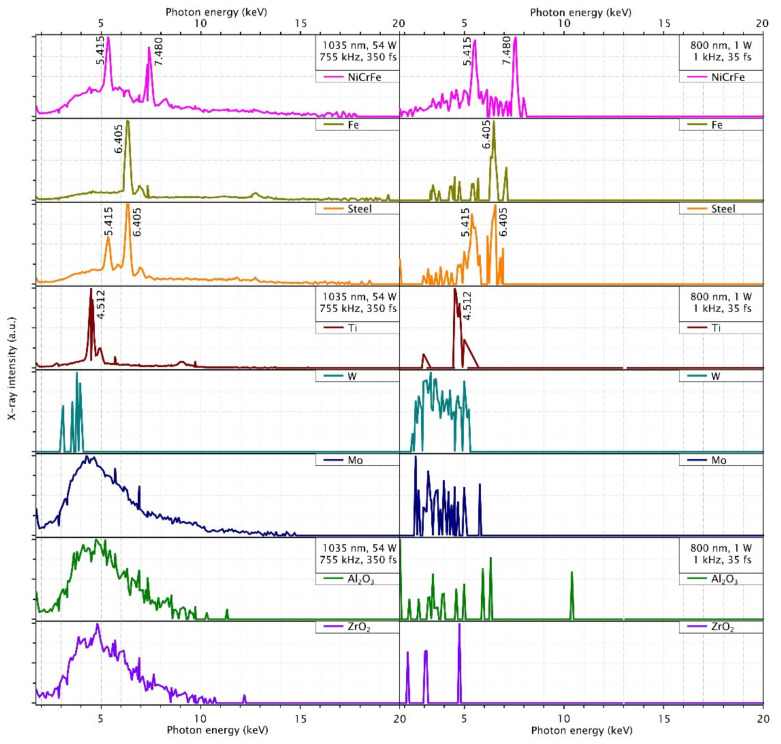
X-ray spectra measured for on the different target material and the incident laser peak intensity listed as NiCrFe and stainless-steel alloys and iron, titanium, tungsten, and molybdenum are investigated using the Silix lambda detector at a distance of 150 mm from the interaction zone in air at an intensity 4.2 × 10^14^ W/cm^2^ (**left**), 2.3 × 10^16^ W/cm^2^ (**right**) respectively for the above mentioned two lasers. The measured spectra of ceramics Al_2_O_3_ and ZrO_2_ are plotted in the same experimental conditions.

This section presents the X-ray spectral distribution for all used materials at the maximum intensity of the KMLabs Dragon and Coherent Monaco laser systems. The Silix lambda spectrodosimeter is used for the spectral measurement and the X-ray detection range is spanned over 2–20 keV. The spectral photon sensitivity of the spectrodosimeter is corrected and calibrated for all 235 channels. In Figure 4 and Figure 5, the spectra are averaged for 6 s and normalized to their maximum values. The X-ray photon counts are higher for the Coherent Monaco laser which is a high repetition rate laser.

The spectral distribution of the plasma emission consists of characteristic lines as well as bremsstrahlung continuum according to the materials interaction with the laser radiation. Here the bremsstrahlung emission is a continuum emission observed below the 5 keV X-ray energy regime. In this intensity regime, the multiphoton ionization (MPI) generates free electrons which become ‘hot’ electrons with high kinetic energy by resonance absorption of photons from the laser pulse. When these hot electrons decelerate, bremsstrahlung emission of X-rays occurs. Increase in X-ray flux can therefore be achieved by increasing the number of hot electrons which are the primary cause for bremsstrahlung [22]. In such a way the bremsstrahlung emission increases with intensity. For the high repetition rate laser, both bremsstrahlung and characteristic line emission are equally probable.

As like the dose rates discussion, the spectral emission of Brass follows similar as its composition of copper but when we see spectral emission of NiCuZn follows the spectral distribution of nickel composition. In addition, the bremsstrahlung emission is also enhanced in the NiCuZn compared to the brass, but it follows the spectral emission of nickel. The spectral emission of NiCrFe follows the chromium and nickel spectral emission not the iron spectral distribution.

For the 755 kHz laser, a broader bremsstrahlung emission is observed in comparison to the 1 kHz laser. Depending on the material, either bremsstrahlung emission or line emission is more dominant. For example, comparing molybdenum with titanium, the dose rate of titanium consists mainly of the characteristic line emission at k-α ≅ 4.512 keV, while the dose rate of molybdenum consists mainly of bremsstrahlung. The characteristic line of molybdenum would be at 17.48 keV.

**Figure 6:**
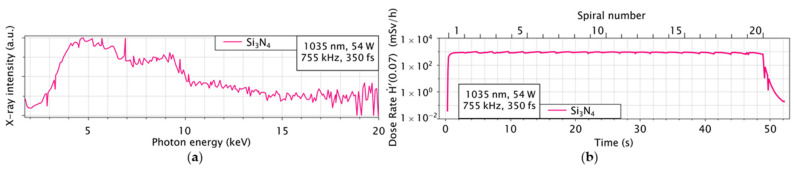
Shown in (**a**) is the spectrum of Si_3_N_4_ measured with the Silix lambda and in (**b**) is the corresponding dose rate over time measured with the OD-02.

In the case of ceramics, the X-ray emission of Si_3_N_4_ is significantly higher compared to other materials shown in Figure 6. It may be due to the nitrides mixed to the silicon. Hence more investigation is necessary to understand the X-ray emission and dose rates on the alloys and ceramics.

X-ray dose attenuation using a shielding material depends on the spectral distribution of the incident X-ray radiation, a shielding factor can only be calculated if the spectral photon flux is exactly known. Hence the spectral distribution of the emitted laser-X-ray radiation is critical for evaluating a protection approach [9]. In addition, the dose rates measured is a combination of bremsstrahlung and characteristic line emission for all the materials.

### 3.3. Discrepency in Dose Rates

This section will take a closer look at the dose rate over time since the laser spot is moving on the material in a spiral format during the material processing. Here, even small changes in laser intensity as well as the position of the focus undergo large effects on the generation of X-rays.

**Figure 7:**
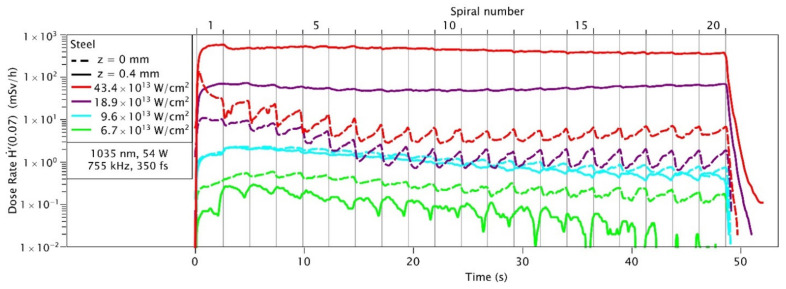
Dose rate Ḣ′(0.07) recorded during different laser processing steps as function of the processing time. The OD-02 dosimeter was placed at a distance of 150 mm from the interaction zone. Each of the individual processing steps is marked by a number on the top of the graph and separated by vertical lines. The laser intensity is 4.2 × 10^14^ W/cm^2^ at a repetition rate 755 kHz and the focal spot diameter 22 µm.

In Figure 7, we see the time-resolved measurement of the dose rate for stainless-steel with Coherent Monaco laser. The process takes about 49 s and consists of 20 spirals. The durations of the spirals are plotted at the top of the plot. The highest dose rate is observed when the laser focus is displaced by ~0.4 mm above the material surface (solid lines in Figure 7). In this situation, the X-ray emission stays at an almost constant level during the entire process duration. When the focus is set at the material surface (dashed lines) the X-ray emission drops after an initial high peak. Additionally, an oscillation is visible which correlates well with the time evolution of the spirals. The strong discrepancy induced by a small shift in focus position is only present at high laser irradiance. In the lower irradiance regime (<10^14^ W/cm^2^) the X-ray emission is strongest when the laser focus is set at the material surface, at an overall lower emission level.

This observation supports the idea that particle and/or plasma shielding strongly affects the X-ray generation for high repetition rate processes at high laser intensities. In this scenario, a slight shift of the focus above the material surface may increase coupling efficiency of laser energy into the ablation plume of previous ablation events, and thus increases the amount of X-ray emission from the re-heated ablation plume. This effect is for high laser intensities most dominant as a large and long living plume can be present. At lower laser intensities the ablation plume is weaker and the effect disappears to a point where the ablation rate of a defocused laser beam is lower resulting in lower X-ray emission compared to the focused beam.

### 3.4. Comparison Silix and OD-02

**Figure 8:**
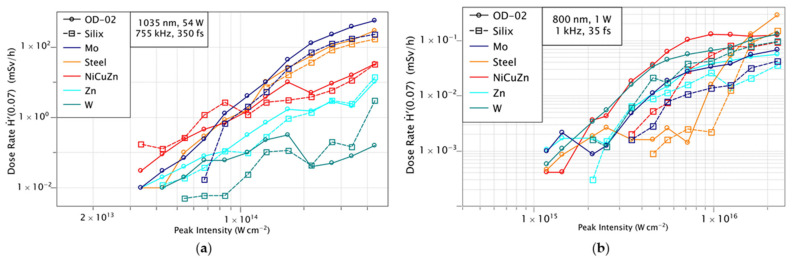
Comparison of the accumulated dose rates using Silix lambda and OD-02 detectors for an intensity range 5 × 10^13^ and 5 × 10^16^ W/cm^2^ by tuning the laser pulse energy for Coherent Monaco (**a**) and KMLabs Dragon laser (**b**). The dotted line represents the dose measurement with the Silix and the solid line the dose measurement with the OD-02.

The X-ray radiation during the micromachining is measured with two detection systems. For the dose rate measurements, the Silix lamda spectrodosimeter is used in addition to a highly sensitive ionisation chamber (OD-02). In addition to the dose rate, the Silix lambda simultaneously measures the spectrum in the X-ray energy range 2–20 keV. The measurement time for the dose rate of the Silix lambda spectrodosimeter is set to 6 s. For the dose rate measurements of the OD-02, the measurement time is 0.078 s. In order to compare both measurements, the measured dose rates shown in Figure 8 were averaged over the whole scanning time.

From the Figure 8, all the materials follow a similar trend. For a comparison of the two detectors, the maximum and average X-ray dose rates from the OD-02 detector are reported in Table 3, together with the X-ray dose rates from the Silix lambda spectrodosimeter for the two laser systems at the respective maximum intensity. The measurements of the two devices are in good agreement. The strong deviation of more than 70% in the case of Si_3_N_4_ can be explained by the spectrum, as the measured X-ray radiation exceeds the measurement range of the Silix lambda spectrodosimeter. However, the X-ray energy regime should be considered while comparing these two detectors. OD-02 is specified 6–100 keV and Silix lambda X-ray spectrometer is specified 2–20 keV. The deviations in the measurements can be explained by the different measuring ranges as well as the spectral radiation depending on laser parameters and the material.

## 4. Conclusions

In conclusion, we have presented a comparison of the emitted X-ray dose rate from laser-driven plasma of several target materials, including pure metals, different alloys, and ceramics using two different ultrafast laser systems.

The X-ray dose rate strongly depends on different laser parameters, e.g., wavelength, pulse duration, repetition rate, and the pulse energy. For a laser intensity above 10^13^ W/cm^2^, all target materials investigated, show a potential hazardous X-ray dose rate which increases with laser intensity. The use of mid-infrared laser wavelengths and multi-kHz repetition rates leads to an additional increase in dose rate compared to standard near-infrared kHz laser systems, even for lower pulse energies. A strong dependency of the dose rate on the laser focus position with respect to the target surface indicates that an additional factor, such as heat accumulation or particle/plasma shielding, may contribute to the X-ray emission during material processing. Such effects are especially relevant in case of multi-pulse irradiation with high repetition rate lasers.

A proper prediction of the X-ray emission remains as a challenge because of many parameters concerning both the laser system and the target, which affect the generation process during laser–material interaction. In order to relate the X-ray dose rate estimation with the spectral information from the laser produced plasma, we have used of a calibrated Silix lambda spectrodosimeter. This detector provides a photon energy spectrum of the X-ray radiation with a dose rate, offering more information about the origin of the X-ray emission below 20 keV. The dose rates from the Silix lambda spectrodosimeter compared with a commonly used OD-02 dosimeter for all the measurements, showing a good agreement between the two detectors.

## Figures and Tables

**Table 1:** Laser parameters used during micromachining.

Laser Parameter	KMLabs Dragon	Coherent Monaco
Wavelength	800 nm	1035 nm
Rep. rate	1 kHz	755 kHz
Pulse energy	1 mJ	72 µJ
Avg. power	1 W	54 W
Pulse duration	35 fs	350 fs
Focus diameter (1/e^2^)	12 ± 2 µm	22 ± 2 µm
Rayleigh length	0.2 mm	0.4 mm
Scanning speed	1.3 mm/s	1000 mm/s
Pulse overlap	10.7 µm	20.7 µm
Polarization	Linear	Linear
Beam profile	Gaussian	Gaussian
Angle of incidence	Perpendicular	Perpendicular
Scanning pattern	20 Spirals	20 Spirals
Scanning time	49 s	49 s

**Table 2:** List of alloys and its percentage of compositions used for the micromachining.

CuZn	Cu 58%	Zn 39%	Pb 3%
NiCuZn	Cu 47–64%	Ni 10–25%	Zn 15–42%
NiCrFe	Ni 72–76%	Cr 18–21%	Fe 5%
Steel 1.4310	Fe 70–77%	Cr 16–19%	Ni 6–9.5%

**Table 3:** Comparison of the measured dose rates from the Silix and the OD-02.

Dose Rate H′(0.07) mSv/h	KMLabs Dragon 2.26 × 10^16^ W/cm^2^			Coherent Monaco 4.22 × 10^14^ W/cm^2^		
	OD-02	OD-02 (Mean)	Silix Lambda	OD-02	OD-02 (Mean)	Silix Lambda
Brass	0.10	0.05	0.09	748	305	171
NiCuZn	0.13	0.05	0.09	248	33	33
Cu	0.15	0.11	0.12	678	229	212
Ni	0.13	0.06	0.08	70	0.77	5.46
Zn	0.06	0.04	0.04	53	10	14
NiCrFe	0.40	0.13	0.30	822	351	215
Steel	0.29	0.09	0.15	432	295	169
Fe	0.08	0.05	0.05	1220	453	237
Mo	0.07	0.06	0.04	745	610	231
Ti	0.05	0.04	0.05	793	166	159
W	0.13	0.12	0.10	72	0.16	2.98
Al_2_O_3_	0.0024	0.0011	-	21	20	10
ZrO_2_	0.0031	0.0007	-	25	18	12
Si_3_N_4_		-	-	988	889	268

## Data Availability

Data sharing is not applicable to this article.
